# Wear Degradation Law of Airport Pavements Under the Coupled Effects of Freeze–Thaw Cycles, Temperature Gradients, and Aircraft Taxiing Loads

**DOI:** 10.3390/ma19071368

**Published:** 2026-03-30

**Authors:** Mingzhi Sun, Xing Gong, Hao Xu, Chuanyu Shao, Zhenyu Zhao

**Affiliations:** 1 Research Institute of Highway Ministry of Transport, Beijing 100088, China; 2 School of Civil Engineering, Chongqing Jiaotong University, Chongqing 400074, China

**Keywords:** airport pavement, wear performance, freeze–thaw cycles, temperature gradient, CNN-BiLSTM

## Abstract

To clarify the wear degradation of airport cement concrete pavements under combined environmental and traffic actions, this study established an environment-tire-pavement multi-physics finite element model incorporating surface texture, freeze–thaw deterioration, temperature gradients, and aircraft lift during taxiing. Indoor rapid freeze–thaw tests, accelerated wear tests, and 3D texture scanning were further conducted to calibrate and validate the model. The results show that temperature gradients significantly amplify pavement wear. At 180 km/h and 1.2 million wear cycles, increasing the temperature gradient from 0 to 60 °C/m increased wear depth and wear mass by about 40% and 96%, respectively. Taxiing speed was negatively correlated with wear, mainly because higher speed reduced tire-pavement contact duration and effective vertical load. Freeze–thaw deterioration was the dominant factor affecting wear, and the coupled freeze–thaw–temperature–load condition produced the most severe damage. The experimental and simulation results agreed well, with R^2^ values above 0.98. Based on the combined experimental-simulation dataset, an interpretable CNN-BiLSTM model was developed for wear-depth prediction, achieving RMSE values of 0.019 and 0.035 for the training and test sets, respectively. SHAP analysis further confirmed that freeze–thaw cycles contributed most to wear prediction. This study can provide a quantitative basis for the wear resistance evaluation, life prediction, and maintenance decision-making of airport pavements.

## 1. Introduction

Cement concrete pavements are widely used in airport pavements due to their high structural strength, excellent durability, and good stability [1,2]. However, during long-term service, with the continuous increase in flight takeoff and landing frequency, the pavement is not only subjected to tire wear during aircraft taxiing but also affected by the coupled influence of complex environmental factors such as temperature variations and freeze–thaw cycles [3,4]. Consequently, its surface functionality and structural performance gradually deteriorate, manifesting as typical distresses including cracks, surface spalling, and potholes, and even leading to severe damage such as brittle fracture and material loosening [5,6,7,8]. Such distresses not only impair the serviceability of the pavement but also adversely affect the safety and stability of aircraft during taxiing, posing a serious threat to aviation operational safety. Therefore, investigating the wear performance and its evolution law of airport pavements holds significant theoretical value and engineering importance for ensuring pavement service performance and extending service life.

Research on the wear mechanism of airport pavements serves as the foundation for revealing their performance evolution laws. Guo et al. [9], based on Persson’s friction theory, established a method for determining the dynamic friction coefficient that considers the three-dimensional texture characteristics of the pavement. By reconstructing the pavement surface topography to obtain contact stress distribution, they provided a theoretical basis for the quantitative evaluation of skid resistance. Sun et al. [10] introduced Miner’s linear cumulative criterion and, combined with the measured lateral distribution pattern of wheel tracks, analyzed the spatial distribution characteristics of pavement damage under dynamic fatigue loading. Yu et al. [11] designed an indoor wear device capable of simulating aircraft tire contact pressure and employed high-precision laser texture scanning technology to acquire surface topography before and after wear. Using indicators such as mean profile depth, bearing area ratio, and British Pendulum Number, the evolution of skid resistance during the wear process was evaluated. The aforementioned studies have laid a solid foundation for understanding pavement wear mechanisms. However, they predominantly focus on the singular effect of load factors, with insufficient consideration given to the coupled influence of environmental factors.

As typical environmental factors, freeze–thaw cycles and temperature gradients significantly influence the microstructure and macroscopic mechanical properties of cement concrete pavements, thereby altering their abrasion resistance [12,13,14]. Under the action of freeze–thaw cycles, the repeated freezing and thawing of pore water within the concrete generates frost heave stress, leading to the initiation and propagation of micro-cracks [15]. Research indicates that with an increasing number of freeze–thaw cycles, the elastic modulus and strength of the concrete gradually deteriorate [16,17]. More importantly, the deteriorating effect of freeze–thaw damage is particularly pronounced on the pavement surface layer: the interfacial transition zone, as the weakest link in concrete, is highly susceptible to debonding between the aggregate and cement matrix under freeze–thaw action. This is the fundamental cause of surface spalling and aggregate exposure, which not only reduces the initial skid resistance of the pavement but also makes aggregate detachment more likely during subsequent wear processes [18,19]. Concurrently, the influence of temperature gradients on pavement performance is manifested in the generation of thermal stresses and their superposition with load-induced stresses. Airport cement concrete pavements, subjected to solar radiation and temperature variations, develop a significant nonlinear temperature distribution along the thickness direction, thereby generating temperature warping stresses and thermal stresses. When a temperature gradient acts in conjunction with aircraft loading, the thermal stress and load stress superimpose, significantly exacerbating fatigue damage on the pavement surface layer [20,21]. Therefore, considering the effects of environmental or load factors in isolation makes it difficult to comprehensively reflect the wear evolution process of pavements under actual operating conditions.

With the continuous development of computational mechanics and numerical simulation methods, multi-physics coupled modeling has found increasingly widespread application in the study of pavement performance. Yu et al. [22] established a finite element model considering the coupling of temperature field and stress field, employing the extended Finite Element Method combined with a modified Paris’ law to simulate the crack evolution process, thereby revealing the damage mechanism of pavement structures under thermo-mechanical coupling. Liu et al. [23] conducted multi-physics coupled analysis using the finite element model, obtaining the relationship between the friction coefficient and tread friction stress under different load, tire pressure, and speed conditions, and subsequently constructed a friction coefficient estimation model. For the grooved concrete structure unique to airport pavements, the introduction of contact wear models has enabled the numerical simulation of surface texture evolution. Kane et al. [24] by constructing a three-dimensional finite element model that considers surface texture characteristics, simulated the contact wear process between the tire and pavement, providing a new approach for the quantitative evaluation of pavement abrasion resistance. However, existing coupled models mostly focus on the coupling between a single environmental factor and load. Simulation studies on pavement wear under the combined action of freeze–thaw, temperature, and load are still relatively rare. In particular, a comprehensive model that simultaneously considers surface texture evolution, freeze–thaw damage accumulation, temperature gradient effects, and aircraft taxiing characteristics has not yet been systematically reported. Considering that airport concrete pavements operate under a unique combination of repeated aircraft loading, high tire contact stress, thermal gradients, and environmental deterioration, particularly under freeze–thaw and temperature-varying environmental conditions, the wear evolution of airport pavements under combined environmental and loading actions remains insufficiently understood. This limitation highlights the necessity of further investigating the multi-factor coupled wear behavior of airport cement concrete pavements.

Addressing the aforementioned research gap, this study aims to systematically investigate the wear evolution law of airport cement concrete pavements under the coupled effects of freeze–thaw cycles, temperature gradients, and aircraft taxiing loads. Unlike previous studies that mainly focused on single-factor loading effects or partial thermo-mechanical coupling, the present study establishes an environment-tire-pavement multi-physics coupled finite element model that simultaneously considers surface texture evolution, freeze–thaw deterioration, temperature gradients, and aircraft lift during taxiing. Using the wear depth in the tire contact area and the wear mass of the wheel track as evaluation indicators, the deterioration characteristics of pavement wear resistance under temperature–load coupling, freeze–thaw–load coupling, and freeze–thaw–temperature–load coupling are analyzed for different temperature gradients and taxiing speeds. Secondly, indoor freeze–thaw cycle tests and accelerated loading wear tests are conducted to obtain the variation laws of material properties and wear indicators under different levels of freeze–thaw action, and the model is validated by comparing the simulation results with the test data. Finally, a prediction sample set is constructed by integrating experimental and simulation data. A CNN-BiLSTM-based wear depth prediction model is proposed, and the SHAP method is utilized for interpretability analysis of the model output to identify the key factors influencing pavement wear performance and their contribution degrees. Therefore, this study offers theoretical and methodological support for abrasion resistance evaluation, residual life prediction, and scientific maintenance decision-making of airport pavements.

## 2. Materials and Experimental

### 2.1. Materials

**Figure 1:**
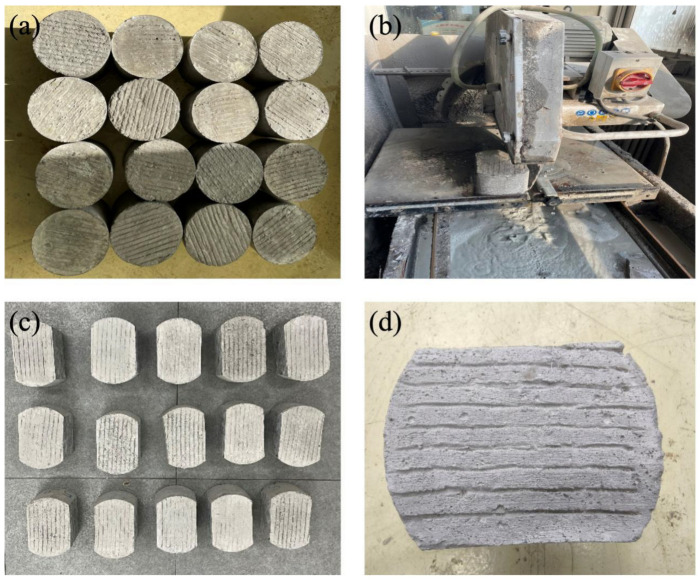
Preparation process and final morphology of the concrete wear specimens: (**a**) Initial grooved concrete specimens, (**b**) cutting of concrete specimens, (**c**) final shaped specimens, (**d**) grooved surface of the final specimen.

According to the determined mix proportion (Table 1), cement concrete specimens with a diameter of 150 mm and a height of 150 mm were prepared. The surface of the specimens was roughened and grooved using a self-made tool, with a groove depth of 3 mm. The prepared specimens were cured for 28 days and then cut with a cutting machine into standard wear test specimens with a height of 100 mm and a width of 100 mm. The preparation process of the specimen is shown in Figure 1.

### 2.2. Indoor Freeze–Thaw Test

Through indoor rapid freeze–thaw cycle tests, the variation patterns of material parameters under freeze–thaw action were obtained, providing parameters for the finite element model in the study and verifying its reliability. All freeze–thaw tests were conducted in an HDK-9 rapid freeze–thaw testing machine (Kexi Instrument and Equipment Co., Ltd, Hebei, China). According to the specification (GB/T 50082-2009) [25], a single freeze–thaw cycle lasted 4 h, with the thawing stage accounting for 25% of the total cycle duration. The number of freeze–thaw cycles was set as follows: 0, 25, 50, 75, 100, 125, 150, 200, 250, and 300 cycles.

### 2.3. Indoor Accelerated Loading Abrasion Test

**Figure 2:**
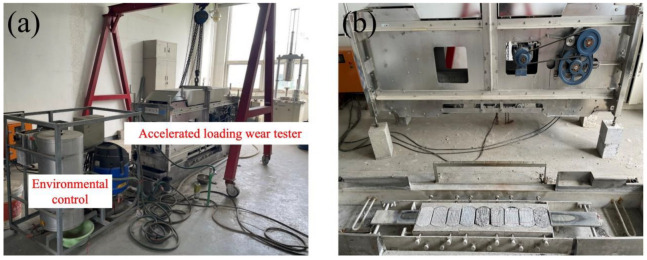
Schematic diagram of indoor accelerated wear testing: (**a**) accelerated loading wear tester, (**b**) specimen installation.

The MMLS3 small-scale accelerated loading testing system was used for wear performance testing (Figure 2). Laboratory-molded cylindrical specimens with basic dimensions of φ150 mm × 100 mm were used in the indoor tests. Each group of tests required the preparation of 9 standard specimens, which were divided into 3 parallel sample groups.

### 2.4. Three-Dimensional (3D) Texture Scanning Test

**Figure 3:**
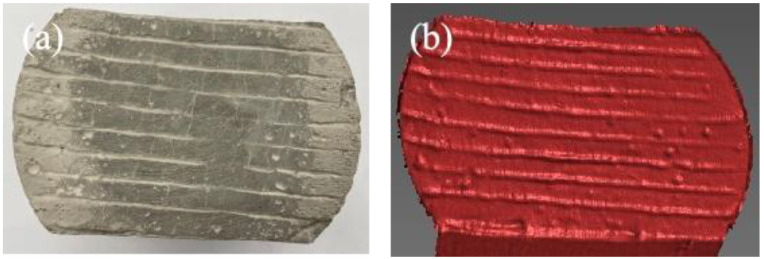
Schematic diagram of the 3D texture scanning test: (**a**) actual surface morphology, (**b**) 3D reconstructed surface morphology.

A three-dimensional scanner (Xtop three-dimensional technology Co., Ltd, Xi’an, China) was used to acquire the point clouds of the specimen surface before and after wear and to reconstruct the texture. The scanning accuracy ranged from 0.01 mm to 0.015 mm, with a scanning dimension of 100–1000 mm. The schematic diagram of the 3D texture scanning test is shown in Figure 3. For each specimen, the surface geometry was scanned before and after abrasion to obtain the corresponding point-cloud datasets. The scanned point clouds were then reconstructed into 3D surface models, and the geometric differences between the pre-wear and post-wear surfaces were used to evaluate the texture degradation and local wear depth.

### 2.5. Construction of an Environment-Tire-Pavement Multi-Phase Coupled Wear Finite Element Model

To characterize the synergistic effects of environmental actions and trafficking loads on airport pavement surface wear, an environment-tire-pavement multi-physics coupled finite element model was developed. A contact wear model was introduced to simulate the evolution of pavement surface texture during aircraft taxiing. In the numerical implementation, ANSYS Workbench 18.2 was used for geometric modeling, local texture construction, and mesh generation of the tire-pavement system, while LS-DYNA was employed to perform tire inflation initialization, thermo-mechanical rolling-contact analysis, and wear simulation. Specifically, the aircraft tire model and the 3D pavement model with grooved surface texture were first established and meshed in ANSYS Workbench and then imported into LS-DYNA for calculation. The pavement temperature gradient was applied layer by layer to obtain a converged temperature and thermal stress field before the tire trafficking simulation was initiated. Subsequently, the rolling contact between the tire and pavement was solved in LS-DYNA, and the Archard wear model was used to calculate the wear depth evolution under coupled freeze–thaw, temperature, and load effects.

#### 2.5.1. Aircraft Tire Model Construction and Material Parameters

According to the literature [26], using the main landing gear of a B737 single-axle dual-wheel assembly as the prototype, a finite element model was established with the H44.5 × 16.5-21 28PR radial tire. To balance computational efficiency and convergence, the geometric structure was simplified by merging the tread rubber and shoulder rubber, applying a filet transition at the interface between the sidewall and the belt layer, retaining the inner liner to ensure airtightness, and using a shared node connection between the rim and the bead to avoid introducing nonlinear contact. During the modeling process, a two-dimensional cross-section of the tire was first drawn and then rotated 360° to generate a 3D geometric model. Subsequently, the model was partitioned into components such as tread rubber, sidewall rubber, inner liner, belt layer, and carcass ply, with corresponding material models and parameters assigned to each. The rated inflation pressure was set at 1.47 MPa, and an airbag model was used to apply the inflation boundary conditions. Based on the rated inflation pressure, the mass of the inflation gas was calculated to be approximately 3.4 kg, which was used to determine the initial state of the airbag. The key material parameters used in the model are all derived from literature [26].

#### 2.5.2. Construction of 3D Model of Cement Concrete Pavement and Boundary Conditions

**Figure 4:**
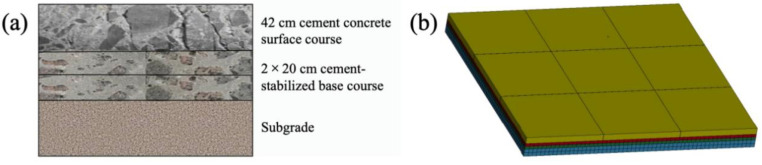
Pavement structure configuration (**a**) and its corresponding finite element model (**b**).

This study adopted a typical rigid pavement structure used in domestic civil aviation airports capable of accommodating large aircraft takeoffs and landings. The specific configuration consists of a 42 cm cement concrete surface layer, a 2 × 20 cm cement-stabilized crushed stone base course, and a subgrade, as illustrated in Figure 4. In accordance with MH/T 5004-2025 “Specifications for airport cement concrete pavement design” [27], nine full-scale concrete slabs, each measuring 5 m × 5 m, were arranged in the surface layer. To mitigate shrinkage and warping stresses induced by temperature variations, reduce stress concentration, and facilitate construction, transverse and longitudinal joints were set between the slabs. Dowel bars are embedded within these joints to enable load transfer between adjacent panels.

**Figure 5:**
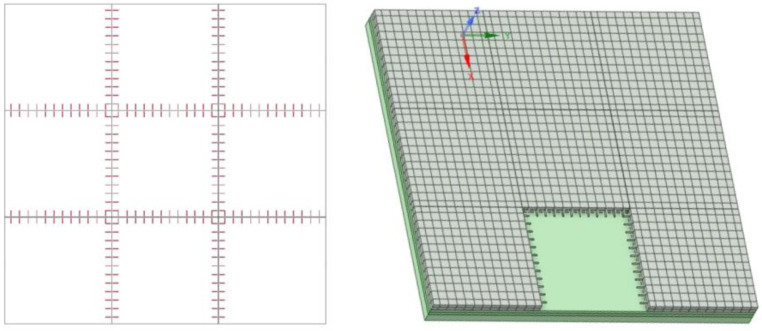
Schematic configuration of built-in dowel bars in the model.

**Figure 6:**
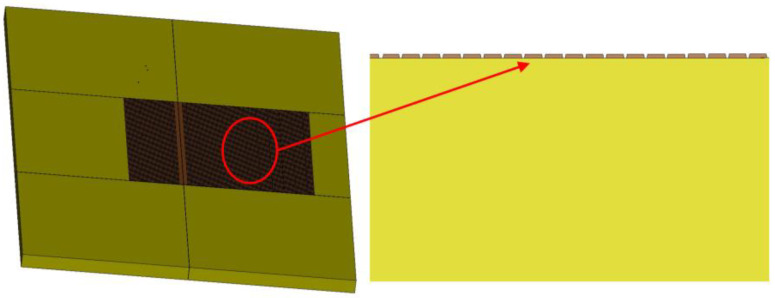
Schematic diagram of the pavement texture structure model.

According to MH/T 5004-2025, “Specifications for airport cement concrete pavement design” [27], dowel bars with a diameter of 38 mm, a length of 550 mm, and a spacing of 380 mm were selected based on the surface layer thickness. The layout of the dowel bars is shown in Figure 5. To investigate the evolution of surface texture under tire action, trapezoidal grooves were designed on the pavement surface following MH/T 5006-2024 “Specifications for construction of aerodrome cement concrete pavement” [28]. The groove geometry features a top width of 6 mm, a bottom width of 4 mm, a depth of 6 mm, and a center-to-center spacing of 38 mm between adjacent grooves. The corresponding model was depicted in Figure 6. Considering computational efficiency, textured areas were only locally arranged on the full-scale pavement, while the remaining regions were modeled as smooth surfaces to reduce the number of elements.

Meshing was performed using hexahedral solid elements, with a mesh size of 5 cm for the surface layer. Non-reflecting boundaries were applied around the model to simulate open boundaries. The bottom of the subgrade was constrained in the Z direction, while lateral displacements in the X and Y directions were restrained. The interlayer friction coefficients were set as 0.8 between the surface layer and the base course and 0.6 between the base course and the subgrade.

#### 2.5.3. Constitutive Model of Airport Pavement Concrete Under Freeze–Thaw Deterioration and Temperature Gradient

First, to describe the deteriorating effect of freeze–thaw cycles on the mechanical properties of the material, an S-shaped decay curve was employed to establish the nonlinear evolution relationship of parameters such as elastic modulus with the number of freeze–thaw cycles N, as shown in Equation (1). Second, to generate a stable temperature stress field, temperature boundaries were applied layer by layer along the pavement thickness direction. The tire trafficking simulation was initiated only after the temperature field and thermal stress field had converged. The temperature gradient as an environmental variable was set to 10 °C/m, 20 °C/m, 40 °C/m, and 60 °C/m, respectively. Finally, in the concrete plastic damage constitutive model, the required axial tensile and compressive strengths were converted from the measured flexural strength values according to GB/T 50010-2010 “Code for design of concrete structures” [29]. The specific material parameters used in the model are from literature [26].

(1) $$P(N)=P_{0}\left(1-\frac{aN}{1+bN^{2}}\right)$$

where *P*(*N*) is the mechanical parameter (dynamic modulus, compressive strength, tensile strength) after *N* freeze–thaw cycles, *P*_0_ is the initial mechanical parameter, *a* is a coefficient controlling the initial decay rate of the mechanical parameter, *b* is a coefficient regulating the subsequent decay magnitude, and *N* is the number of freeze–thaw cycles.

#### 2.5.4. Taxiing Model Considering Aircraft Lift

Owing to the larger curvature of the aircraft wing’s upper surface, the airflow accelerates, resulting in lower pressure, while the airflow on the lower surface is decelerated due to obstruction, leading to higher pressure. This pressure difference generates lift. Simultaneously, the convergence of airflow at the trailing edge of the wing creates downward momentum and vortex effects, further enhancing lift generation. During the taxiing phase on the airport runway, the aircraft load is jointly governed by gravity, lift, and pavement reaction forces, with the mechanical relationship expressed in Equations (2) and (3). As the lift coefficient is negligible at low taxiing speeds (e.g., 40 km/h), it can be disregarded. However, as speed increases (100 km/h, 180 km/h), lift significantly rises, resulting in a reduction in the effective load exerted by the tires on the pavement. Therefore, speeds of 40 km/h, 100 km/h, and 180 km/h are selected as representative velocities for low-speed, medium-speed, and high-speed taxiing phases, respectively. Additionally, considering the impact of fuel consumption during flight, the actual landing load is lower than the design value. In the model, the weight of the B737-800 aircraft is taken as 664 kN, with a main landing gear distribution coefficient of 0.95.

(2) $$p_{v}=G-R_{y}$$

(3) $$R_{y}=C_{y}\rho S\frac{v^{2}}{2}$$

where *P_v_* is the load on the aircraft during taxiing, *R_y_* is the aircraft lift, *v* is the taxiing speed, *ρ* is the air density, *C_y_* is the lift coefficient, and *S* is the lift area.

#### 2.5.5. Environment-Tire-Pavement Multi-Phase Coupled Wear Interaction Model

The Archard wear model is one of the most classic wear models in the field of mechanical tribology. It is used to describe the relationship between the volumetric wear rate of two contacting surfaces and the contact pressure, sliding distance, and material properties. The wear coefficient in the model characterizes the degree of surface material wear or spalling of concrete under external pressure or friction, serving as an important indicator of the abrasion resistance of concrete materials [30]. The formula for the Archard wear model is shown in Equation (4). During the concrete wear process, due to the adhesion of rubber particles on the textured surface, errors may occur in the volume loss of concrete in the reconstructed texture model. To make the magnitude and distribution of damage more reasonable, it is necessary to transform the Archard model. The expression for wear depth after transformation is given in Equation (5).

(4) $$V=K\frac{F\cdot s}{H}$$

(5) $$h=K\frac{N\cdot d}{H}$$

where *V* is the wear volume of the object (m^3^), *K* is the dimensionless wear coefficient, taken as 9.5793 × 10^−7^, *s* is the relative sliding distance of the object (m), *H* is the Brinell hardness of the material (N/mm^2^), *F* is the normal force at the two-phase contact interface (N), *h* is the wear depth (m), *N* is the normal contact pressure (MPa), and *d* is the relative sliding distance (m).

### 2.6. Convolutional Neural Network-Bidirectional Long Short-Term Memory (CNN-BiLSTM)

**Figure 7:**
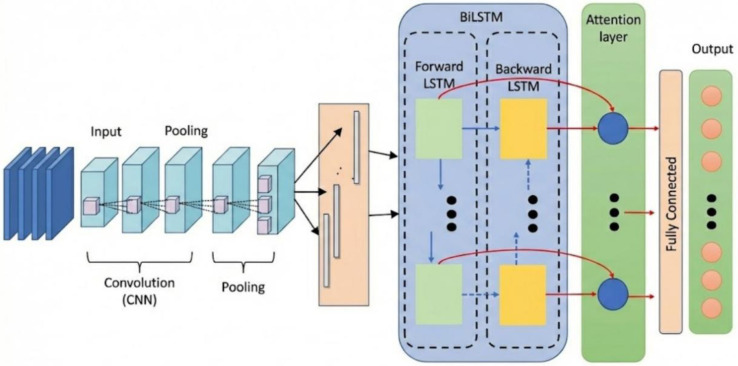
Architecture diagram of the CNN-BiLSTM model.

To systematically investigate the factors influencing wear performance, this study considers multiple variables, including taxiing load (represented by dynamic load coefficients of 1.25, 1.5, and 2.0), number of wear cycles (ranging from 40,000 to 1.2 million), freeze–thaw cycles (25, 50, 75, 100, 150, and 200), and temperature gradient. Due to the negligible effect of low temperature gradients on pavement wear performance, the analysis focuses on gradients between 30 °C/m and 60 °C/m. Based on simulation data combined with indoor accelerated loading test results, a prediction dataset for pavement wear resistance is constructed. The input features include dynamic load coefficient, number of wear cycles, freeze–thaw cycles, and temperature gradient, while the output is wear depth. Notably, the number of wear cycles reflects the temporal evolution characteristics of wear and serves as a key variable for constructing sequence prediction models. Before model training, all samples were normalized and divided into training and test sets. Subsequently, convolutional neural networks were employed to extract local variation features from the multi-factor input data, and a bidirectional long short-term memory network was used to capture both short-term fluctuations and long-term evolution trends in pavement wear from forward and backward directions. Finally, the predicted wear depth was generated through a fully connected layer, and root mean square error was adopted to evaluate model performance [31,32], The architecture diagram of CNN-BiLSTM modle is shown in Figure 7.

### 2.7. SHAP Global Interpretation

To explain the contribution of each influencing factor to the prediction results, the SHAP model interpretation framework based on cooperative game theory was adopted to quantify the contribution of each feature to the model’s prediction outcomes. The calculation formula for the Shapley value of feature *i* is shown in Equation (6) [33].

(6) $$\phi_{i}(f)=\sum_{S\subseteq N\backslash \{i\}}\frac{|S|!(|N|-|S|-1)!}{|N|!}\left(f_{S\cup \{i\}}-f_{S}\right)$$

where *N* is the set of all features, ∣*S*∣ is the number of features in the subset, and ∣*N*∣ is the total number of features.

## 3. Results and Discussion

### 3.1. Pavement Wear Resistance Under Temperature–Load Coupling

**Figure 8:**
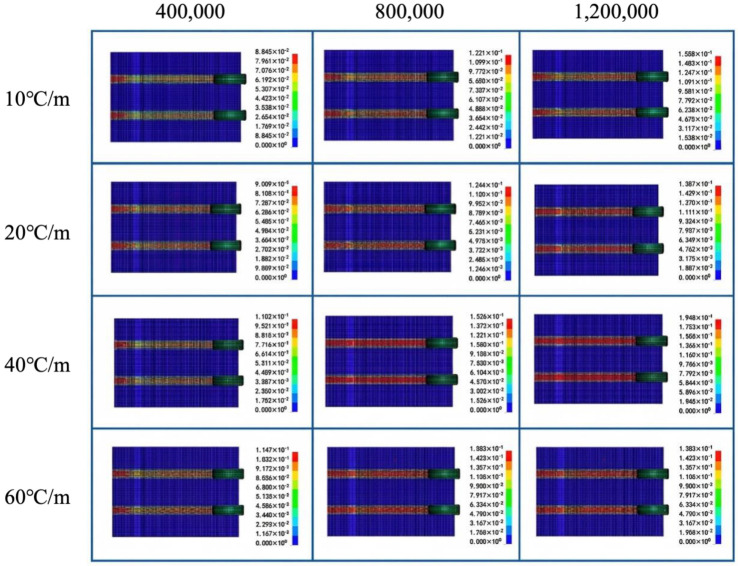
Variation in wear depth under temperature–load coupling at a taxiing speed of 180 km/h.

Using the wear depth of the pavement element in the tire center area and the wear mass of the single-wheel track within a 5 m length as evaluation indicators, the degradation law of pavement wear resistance under the coupled effect of temperature and load was revealed. Figure 8 shows a cloud map of pavement wear depth under typical working conditions with the coupled temperature–load effect (taking 180 km/h as an example). It can be observed from the figure that wear is mainly concentrated in the contact area between the aircraft tire and the pavement, forming two distinct wear bands along the aircraft taxiing direction. The wear depth exhibits a significant gradient distribution, with the deepest wear occurring in the middle of the tire contact area (red region), gradually transitioning to a light blue region towards the edges, indicating minimal wear. This distribution pattern indicates that the dynamic loading of the pavement by the tire is the primary cause of wear, while also reflecting the regular variation in wear with tire pressure distribution.

**Figure 9:**
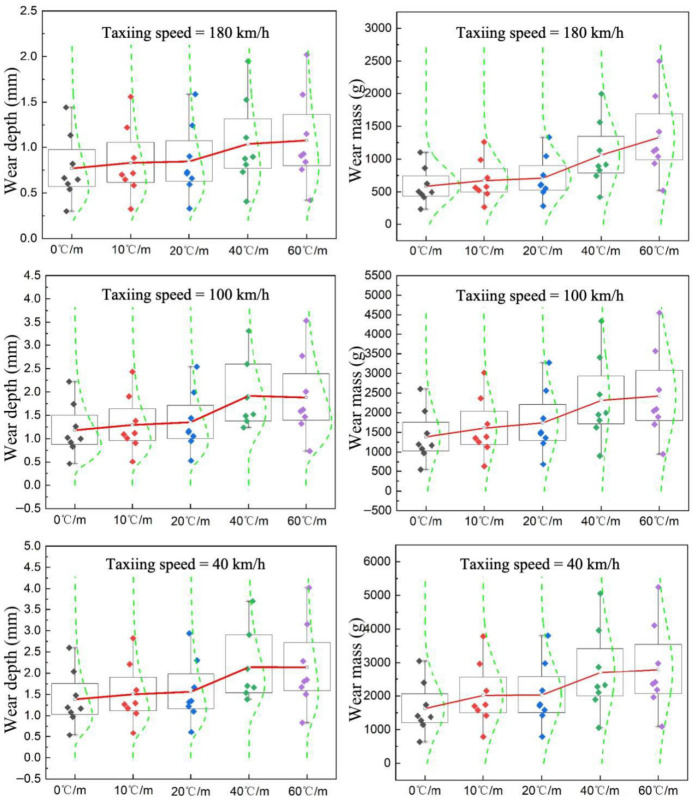
Variation in pavement wear resistance under temperature–load coupling.

To further analyze the influence of the coupled temperature–load effect on pavement wear resistance at different taxiing speeds, data charts showing the variation in pavement wear depth and wear mass within the 5 m wheel track over time under coupled temperature gradient and load conditions at different taxiing speeds were plotted, as shown in Figure 9 (taking 180 km/h as an example). The maximum wear depths at 180 km/h, 100 km/h, and 40 km/h are 2.02 mm, 3.53 mm, and 4.02 mm, respectively; the corresponding maximum wear masses are 2159 g, 4552 g, and 5245 g. The maximum wear depths at 180 km/h, 100 km/h, and 40 km/h are 2.02 mm, 3.53 mm, and 4.02 mm, respectively; the corresponding maximum wear masses are 2159 g, 4552 g, and 5245 g. These results are consistent with previous airfield-pavement evaluation studies and aircraft taxiing analyses, which indicate that the wings continue to provide lift during the landing roll and taxiing stage, thereby reducing the effective wheel load transmitted to the pavement, and that the lift force increases with taxiing speed for the same aircraft type [34,35].

Under the coupled effect of temperature gradient and load, both wear depth and wear mass show an increasing trend with the number of wear cycles across all temperature gradient conditions, and the values increase with the temperature gradient. To define the wear-evolution stages more quantitatively, the wear depth-cycle curves were interpreted according to their slope-change characteristics. Based on the variation in curve growth rate, the wear process can be divided into three stages: the rapid wear stage (40,000 to 120,000 wear cycles), the stable wear stage (160,000 to 400,000 wear cycles), and the accelerated fatigue wear stage (800,000 to 1.2 million wear cycles). In the rapid wear stage, the wear depth curves under each temperature gradient exhibit approximately linear growth, indicating rapid removal of the surface mortar layer. In the stable wear stage, the curve growth becomes gentler and the wear rate decreases, suggesting that the initial mortar wear gradually stabilizes. In the accelerated fatigue wear stage, the curve rises sharply again, which is attributed to the superposition of thermal stress and load-induced stress, as well as intensified interfacial damage and aggregate detachment. Under the same number of wear cycles, pavement wear under high-temperature gradient conditions is significantly greater than that under low-temperature gradient conditions. Taking 1.2 million wear cycles at a taxiing speed of 180 km/h as an example, compared with the single load condition (temperature gradient of 0), the wear depth increases by 40%, 35%, 10%, and 8% under temperature gradients of 60 °C/m, 40 °C/m, 20 °C/m, and 10 °C/m, respectively, while the wear mass increases by 96.0%, 80.9%, 21.0%, and 14.5%. The effect of temperature on wear is minimal under lower temperature gradients. This indicates that the presence of a temperature gradient accelerates the wear of concrete pavements, which is attributed to the superposition of thermal stress induced by the temperature gradient and mechanical stress induced by loading, significantly aggravating the degree of wear. The present results confirm that temperature gradients do not merely affect structural stress states but also accelerate surface wear evolution. This observation is consistent with previous thermo-mechanical studies showing that temperature gradients increase pavement warping stress and amplify load-induced responses [2]. However, unlike earlier studies that mainly focused on stress, strain, or structural response, the present work further demonstrates how temperature gradients translate into measurable surface wear depth and wheel-track mass loss under rolling aircraft loads.

**Figure 10:**
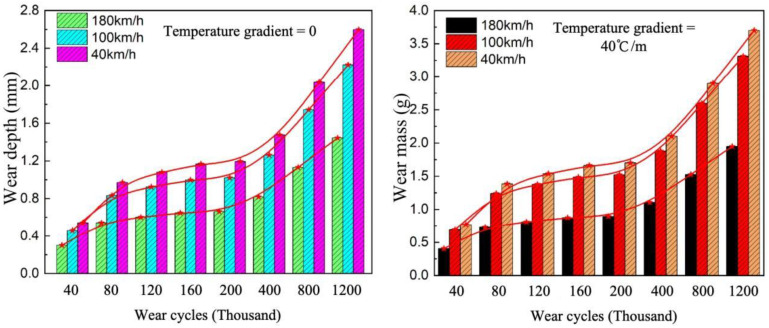
Variation in wear depth at different taxiing speeds.

Selecting temperature gradients of 0 and 40 °C/m and using wear depth as the evaluation indicator, the influence of different taxiing speeds on pavement wear performance was analyzed. As shown in Figure 10, under the same number of wear cycles, a higher taxiing speed corresponds to a lower wear depth, exhibiting a clear negative correlation. Wear depth is highest under low-speed taxiing conditions and gradually decreases as speed increases. This suggests that under high-speed taxiing conditions, the shorter contact time between the tire and the pavement, combined with the reduced load per unit area on the pavement due to aircraft lift, results in a lower degree of wear.

### 3.2. Pavement Wear Resistance Under Freeze–Thaw–Load Coupling

**Figure 11:**
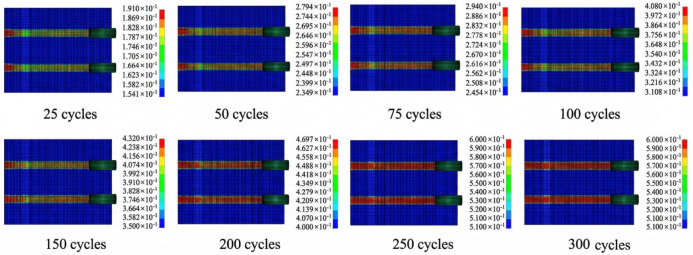
Variation inof wear depth under freeze–thaw–load coupling.

**Figure 12:**
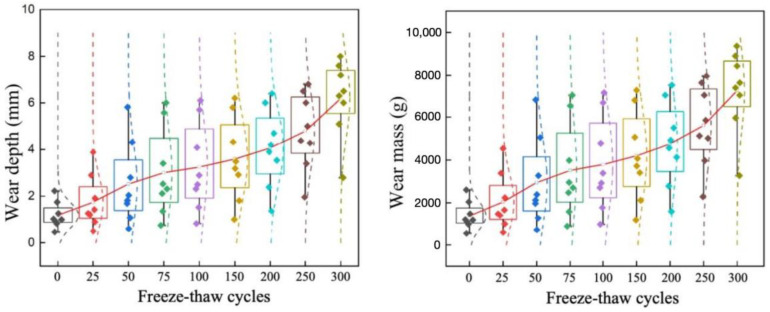
Variation trend of pavement wear performance under freeze–thaw–load coupling.

Figure 11 presents a cloud map of wear depth variation under different numbers of freeze–thaw cycles at 400,000 wear cycles. Meanwhile, using wear mass and wear depth as indicators, the trend of variation across all working conditions is illustrated in Figure 12. It can be observed that under the same number of wear cycles, as the number of freeze–thaw cycles increases, the wear depth continuously increases, and the rate of increase also accelerates. When the number of freeze–thaw cycles is less than 100, the increase in wear depth is relatively gentle, with small differences among various freeze–thaw cycle conditions. At this stage, the surface texture of the concrete has not been completely damaged, so the impact of freeze–thaw cycles is relatively limited. However, after exceeding 200 freeze–thaw cycles, the difference in wear depth gradually widens. The pronounced increase in wear after repeated freeze–thaw cycling can be explained from a microstructural perspective. Freeze–thaw action promotes the initiation of internal microcracks, accelerates crack propagation, and weakens the interfacial transition zone (ITZ) between aggregate and mortar. As the surface mortar becomes progressively loosened, aggregate-matrix debonding and aggregate pull-out become more likely under repeated tire loading, which in turn accelerates surface material removal and groove deterioration. This mechanism is consistent with previous studies on freeze–thaw-induced cracking and ITZ deterioration in concrete materials [36].

### 3.3. Pavement Wear Resistance Under Freeze–Thaw–Temperature–Load Coupling

**Figure 13:**
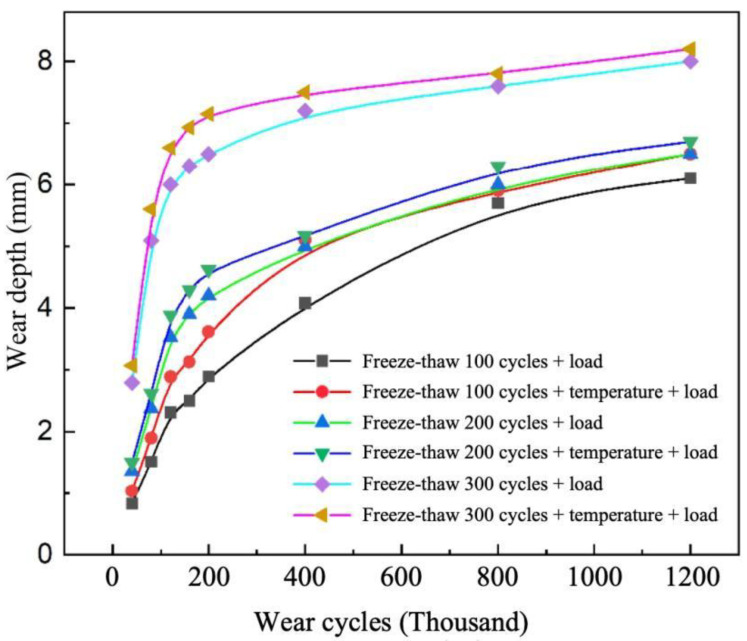
Variation trend of pavement wear performance under temperature–freeze–thaw–load coupling.

**Figure 14:**
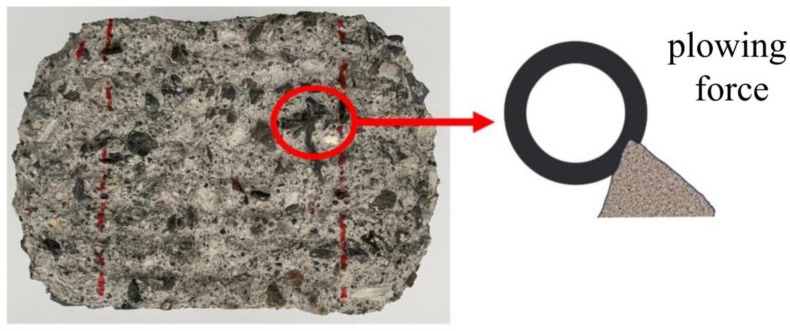
Schematic diagram of tire cutting action on pavement.

The degradation law of pavement wear resistance under the coupled effects of temperature (40 °C/m), freeze–thaw cycles (100, 200, and 300 cycles), and loading at a taxiing speed of 100 km/h is shown in Figure 13. It can be observed that under the coupled effect of temperature, freeze–thaw, and loading, the maximum wear depth reaches approximately 8 mm, while under the coupled freeze–thaw and loading condition, the maximum wear depth is about 7 mm. The wear depth resulting from the triple coupling effect is significantly higher than that under the freeze–thaw–load coupling alone, indicating that the superposition of freeze–thaw cycles and the temperature–load coupling effect causes more severe damage to the concrete surface, leading to a substantial decline in material wear resistance. This effect is particularly pronounced under high numbers of freeze–thaw cycles, where the cumulative impact becomes even more significant. The influence of freeze–thaw action on the abrasion resistance of cement concrete pavements manifests in two primary aspects. On one hand, freeze–thaw cycles degrade the material properties of concrete, leading to the formation and propagation of micro-cracks within the concrete matrix. This results in surface spalling and a reduction in material strength. Simultaneously, freeze–thaw action disrupts the bonding force between the cement matrix and aggregates, causing aggregates to become prone to detachment or displacement after freeze–thaw cycles, thereby diminishing the abrasion resistance of the concrete. On the other hand, after aggregates become exposed, the pavement surface becomes increasingly irregular due to the loss of surface smoothness. This intensifies the micro-cutting action of surface protrusions and increases the plowing force in localized areas, as illustrated in Figure 14. Consequently, during aircraft taxiing, the frictional force generated at the tire-pavement interface increases, thereby accelerating the wear of the concrete surface. The increase in plowing force means that the grooved surface is subjected to greater mechanical impact from sliding loads, which exacerbates the detachment and friction of both aggregates and the cement matrix, ultimately reducing the pavement’s abrasion resistance.

### 3.4. Validation of Airport Pavement Wear Model Based on Indoor Tests

**Figure 15:**
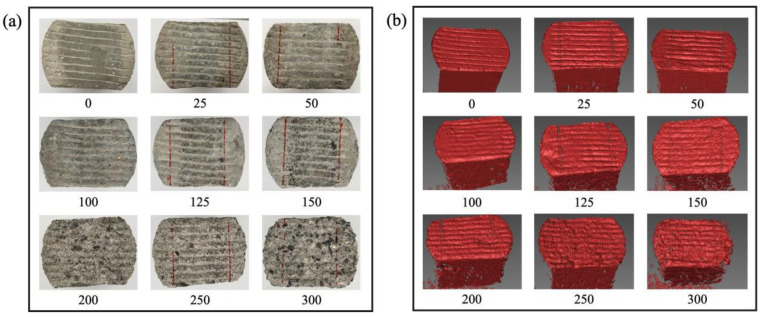
Surface texture structure of specimens after 500,000 wear cycles under different freeze–thaw cycles: (**a**) actual surface morphology, (**b**) 3D reconstructed surface morphology.

Indoor tests were conducted to validate the coupled freeze–thaw–load wear model. Figure 15 shows the surface texture of specimens subjected to different numbers of freeze–thaw cycles, scanned using a 3D texture scanner. As can be observed from Figure 15, after 500,000 wear cycles, the surface texture of the specimens gradually becomes rougher with an increasing number of freeze–thaw cycles. For specimens not subjected to freeze–thaw cycles, the groove texture on the surface remains clear and regular, with no significant signs of wear, and the texture depth and integrity are well maintained. After 25 to 50 freeze–thaw cycles, the edges of the grooves begin to dull, slight wear marks appear on the surface, and the texture depth decreases, although the overall outline remains visible. At this stage, the surface mortar layer is progressively weakened, but large-scale aggregate exposure has not yet occurred. After 100 to 150 freeze–thaw cycles, surface wear intensifies, the grooves become increasingly indistinct, and textures in some areas nearly disappear. The mortar layer is almost completely worn away, and coarse aggregates begin to become exposed. The synergistic effect of freeze–thaw cycles and wear accelerates surface layer damage. During this stage, wear damage is primarily concentrated on the surface layer and extends to deeper structural levels. After 200 to 300 freeze–thaw cycles, the grooves disappear entirely, and a large area of aggregates becomes exposed on the surface. The original texture of the specimen is completely destroyed, and the surface is no longer smooth, exhibiting a distinctly rough, granular appearance. The bonding interface between aggregates becomes loose. At this stage, crack propagation caused by freeze–thaw cycles and the separation of aggregate interfaces are significant, leading to severe surface spalling. As the number of wear cycles increases, the degree of surface damage stabilizes, although the aggregate layer continues to be eroded.

**Figure 16:**
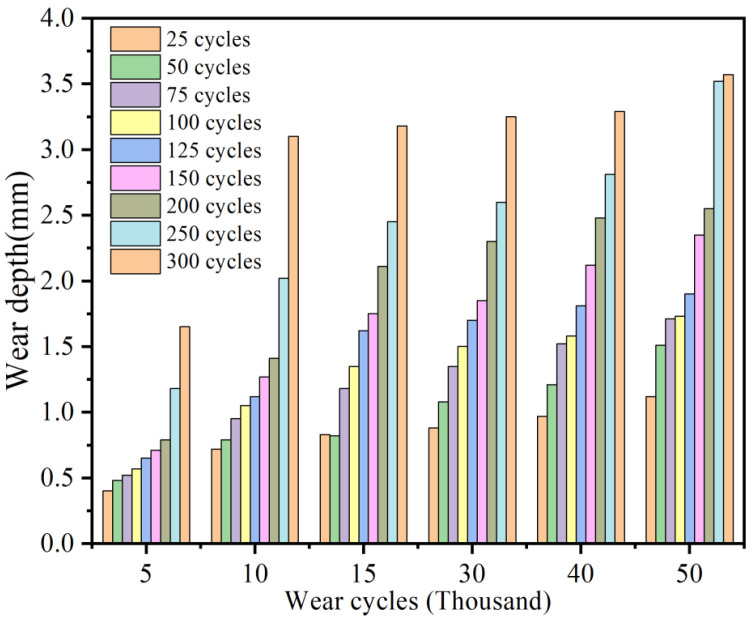
Comparison of wear depth results between model calculation and indoor test under freeze–thaw–load coupling.

**Figure 17:**
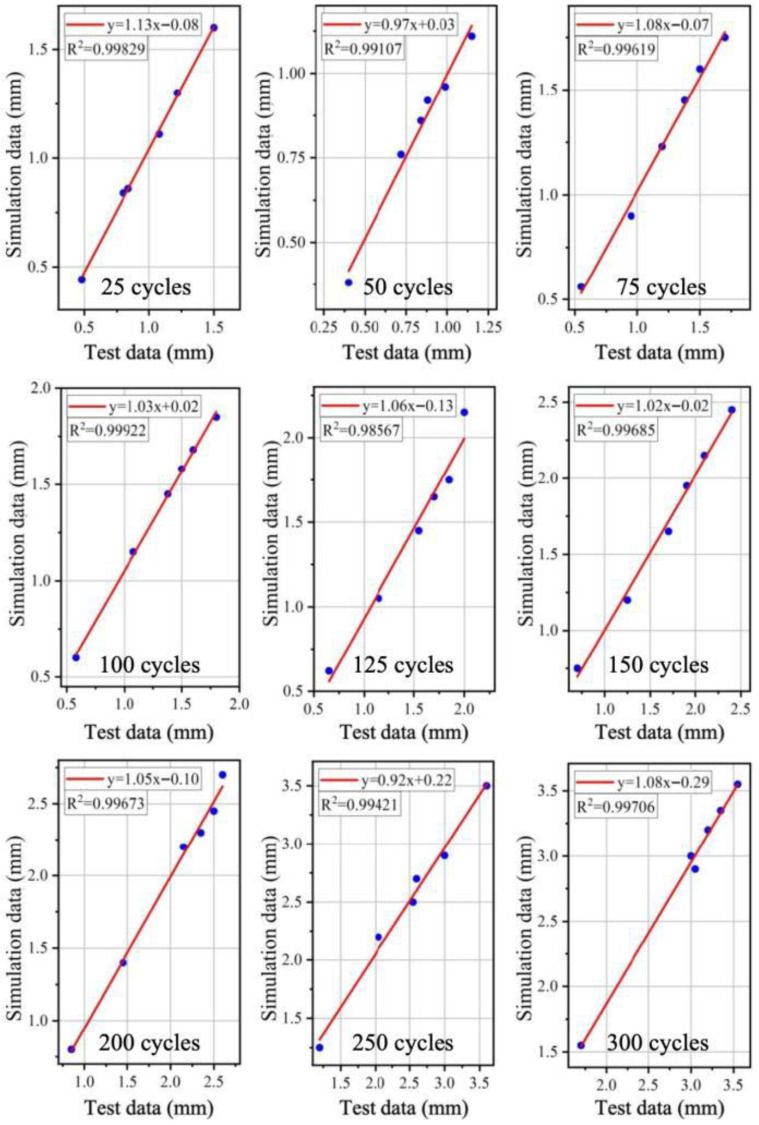
Fitting results of wear depth from model calculation and indoor test.

To quantify the results of the indoor tests, the variation in wear depth with the number of wear cycles under the coupled freeze–thaw–load action was plotted, as shown in Figure 16. It can be observed that there is a significant positive correlation between wear depth and the number of wear cycles, and the trends of the wear depth curves from the tests and the model simulations are consistent. Fitting analysis was conducted on the test data and simulation data (Figure 17), revealing that the R^2^ values for different data groups are all greater than 0.98. This indicates an excellent fit between the experimental values and the model simulation values, demonstrating that the established model can accurately represent the actual working conditions. Although the agreement between experiments and simulations is satisfactory, some discrepancies are still unavoidable. These may arise from the assumption of material homogeneity in the numerical model, the use of a constant wear coefficient, the difference between laboratory-scale loading and full-scale aircraft taxiing conditions, possible measurement errors in 3D texture reconstruction, and the scale effect of the specimens.

### 3.5. Prediction of Cement Pavement Wear Resistance Based on CNN-BiLSTM

**Figure 18:**
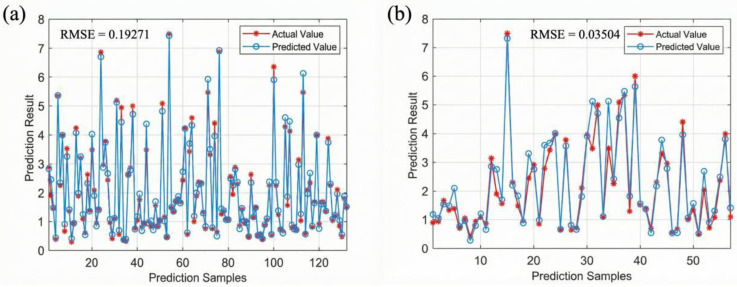
RMSE results of predicted and actual values: (**a**) training set, (**b**) test set.

Figure 18 presents the simulation results of the pavement wear resistance prediction model considering the coupled effects of multiple factors. It can be observed that the RMSE values for the model training set and prediction set are 0.019 and 0.035, respectively. Both the training set and prediction set exhibit relatively small errors when comparing the predicted results with the actual values. The model effectively captures the data variation trends and demonstrates high prediction accuracy, indicating that CNN-BiLSTM can reliably predict the wear resistance of pavements under the coupled effects of multiple factors.

### 3.6. Multi-Factor Synergistic Analysis of Wear Resistance Based on SHAP Global Interpretation

**Figure 19:**
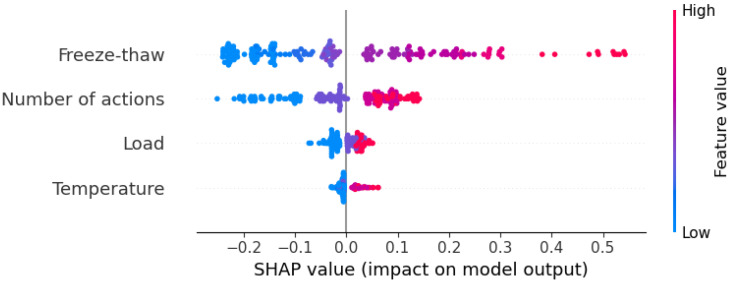
Overall summary plot of pavement wear resistance under environment–load coupling.

Figure 19 presents a SHAP value-based overall feature contribution plot for pavement wear resistance under the coupled effects of multiple factors, reflecting the average impact of each input feature on the model output. Figure 19 illustrates the interaction effects among these factors. As shown in Figure 18, freeze–thaw cycling is the most significant factor affecting pavement wear resistance, with the highest absolute SHAP value, indicating that freeze–thaw action is the dominant factor leading to the deterioration of pavement wear resistance. The second most influential factor is the number of wear cycles, where the pavement surface gradually deteriorates with increased usage, resulting in a continuous decline in wear resistance. In contrast, the SHAP values for load and temperature are relatively low, suggesting that, within the range of conditions considered in this study, their direct impact on wear performance is limited. Notably, when the number of freeze–thaw cycles is high, the corresponding SHAP value is also high, indicating that the deteriorating effect of freeze–thaw is further amplified under severe freeze–thaw conditions. Similarly, an increase in the number of wear cycles corresponds to a rise in SHAP value, reflecting the cumulative damage effect of the material during long-term service. Regarding load, as the tire pressure remains constant in the model, its influence on wear is primarily reflected in structural fatigue damage rather than being a direct driver of surface wear.

**Figure 20:**
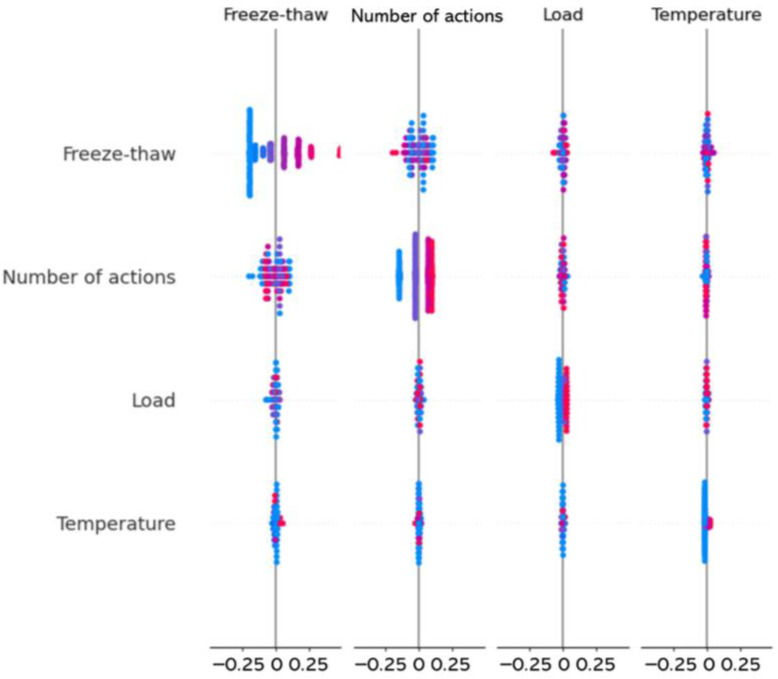
Multi-factor interaction analysis plot.

Figure 20 further reveals the interaction characteristics among the influencing factors. The interaction between freeze–thaw cycling and the other factors is particularly prominent, indicating that freeze–thaw action not only directly reduces pavement wear resistance but also enhances the deterioration effects of loading and wear cycles. In contrast, the interaction between temperature gradient and the other factors is relatively weak, with SHAP values mostly concentrated near zero, indicating a smaller direct contribution within the temperature–gradient range considered in this study. Combined with the results in Section 3.1, Section 3.2 and Section 3.3, these findings suggest that freeze–thaw deterioration is the dominant factor governing pavement wear, whereas temperature gradient mainly acts as an amplifying factor under already weakened pavement conditions. Moreover, the higher wear depth under freeze–thaw–temperature–load coupling than under freeze–thaw–load coupling indicates a synergistic amplification tendency rather than a purely additive effect. Therefore, freeze–thaw cycling is the core factor affecting pavement wear resistance, while its interaction with loading and temperature accelerates pavement deterioration.

## 4. Conclusions

By integrating laboratory testing, multi-physics coupled finite element simulation, and interpretable data-driven prediction, this study investigated the wear evolution of airport cement concrete pavements under the coupled effects of freeze–thaw action, temperature gradient, and aircraft taxiing load. The main conclusions are summarized as follows.

1. Under the coupled effect of temperature and load, the temperature gradient has a significant amplifying effect on wear, and this effect becomes more pronounced as the gradient increases. At a speed of 180 km/h and after 1.2 million wear cycles, compared with the condition without a temperature gradient, increasing the temperature gradient from 0 °C/m to 60 °C/m increases the wear depth and wear mass by approximately 40% and 96%, respectively.
2. Taxiing speed has a significant negative correlation with pavement wear, with high-speed taxiing reducing the degree of wear. At a temperature gradient of 40 °C/m, the maximum wear depths at taxiing speeds of 180 km/h, 100 km/h, and 40 km/h are 2.02 mm, 3.53 mm, and 4.02 mm, respectively.
3. Freeze–thaw deterioration is the dominant factor affecting pavement wear resistance. When temperature gradient, freeze–thaw action, and load are superimposed, wear reaches its maximum, with a maximum wear depth of approximately 8 mm, which is higher than the 7 mm observed under freeze–thaw–load coupling conditions. The interaction of freeze–thaw, temperature, and load exhibits a synergistic amplification tendency rather than a purely additive effect.
4. The wear trends obtained from indoor freeze–thaw cycle tests and accelerated loading wear tests show a high degree of agreement with the simulation results, with the fitting degree of all wear indicators exceeding 0.98. This verifies that the established environment-tire-pavement multi-physics coupled finite element model has high accuracy in describing wear evolution and quantifying wear indicators.
5. After constructing a prediction sample set by integrating experimental and simulation data, the proposed CNN-BiLSTM wear-depth prediction model demonstrates high predictive capability, with RMSE values of 0.019 and 0.035 for the training set and test set, respectively. SHAP interpretability analysis further indicates that freeze–thaw cycles contribute the most to pavement wear prediction, which is consistent with the experimental and simulation results.

This study can provide a quantitative basis for abrasion resistance evaluation, residual life prediction, and scientific maintenance decision-making for airport pavements. Future work will focus on field-scale validation and on incorporating additional service factors, such as moisture condition, variable wear coefficients, and different aircraft types, to further improve the practical applicability of the proposed framework.

## Figures and Tables

**Table 1:** Cement concrete mix proportion.

Water–Cement Ratio	Cement (kg/m^3^)	Water (kg/m^3^)	Aggregate (kg/m^3^)	Sand (kg/m^3^)	Water Reducer (kg/m^3^)	28-Day Flexural Strength (MPa)
0.41	424	174	1190	591	2.5	5.82

## Data Availability

The original contributions presented in this study are included in the article. Further inquiries can be directed to the corresponding author.

## References

[1] Jamieson S., White G. (2025). Parametric Analysis of a Dowelled Construction Joint for Rigid Aircraft Pavement Load Transfer Using Finite Element Methods. Int. J. Pavement Eng..

[2] Li M., Zhang W., Wang F., Li Y., Liu Z., Meng Q., Huo F., Zhao D., Jiang J., Zhang J. (2024). A State-of-the-Art Assessment in Developing Advanced Concrete Materials for Airport Pavements with Improved Performance and Durability. Case Stud. Constr. Mater..

[3] Zhu G., Zhang D., Zhang L., Xu J., Guo B., Tan Y. (2025). Research on Multi-Scale Damage Behavior and Structural Evolution of Hardened Cement Paste by High-Power Nanosecond Pulsed Laser: Based on Laser Flux Range in Airport Pavement Engineering. Constr. Build. Mater..

[4] Guo Y., Zhao J., Hao T., Sun Q. (2025). Degradation Behavior of Surface Wear Resistance of Marine Airport Rigid Pavements. Materials.

[5] Liu S.-F., Ling J.-M., Zhu L.-G., Li P.-L., Lin S. (2026). A Novel Analytical Approach for the Spatial Distribution of Accumulated Damage on the Airport Runway Considering Dynamic Fatigue. Eng. Fail. Anal..

[6] Wang X., Ma Z., Hu X., Cao X., Dong Q. (2025). Void Detection of Airport Concrete Pavement Slabs Based on Vibration Response Under Moving Load. Sensors.

[7] Ahmad F., Jamal A., Iqbal M., Alqurashi M., Almoshaogeh M., Al-Ahmadi H.M., E. Hussein E. (2022). Performance Evaluation of Cementitious Composites Incorporating Nano Graphite Platelets as Additive Carbon Material. Materials.

[8] Ahmad Z., Qureshi M.I., Ahmad F., El Ouni M.H., Asghar M.Z., Ghazouani N. (2025). Effect of Macro Synthetic Fiber (MSF) on the Behavior of Conventional Concrete and the Concrete Containing e-Waste Aggregates. Mater. Struct..

[9] Guo W., Huang K., Xiong J., Jiang R., Ming Y. (2026). Determination of Pavement Texture Parameters for Persson’s Friction Theory Based on Wear Evolution Considerations. Tribol. Int..

[10] Sun L., Hudson W.R. (2005). Probabilistic Approaches for Pavement Fatigue Cracking Prediction Based on Cumulative Damage Using Miner’s Law. J. Eng. Mech..

[11] Yu Y., Wang H., Crispino M., Li Y., Ketabdari M., Xu G., Yang J. (2025). Wear Behavior and Skid-Resistance Durability of Runway Pavements Based on Surface Texture Characteristics. Tribol. Int..

[12] Tao C., Dong L., Suo M. (2025). Study on the Impact of Combined Action of Temperature Differential and Freeze–Thaw Cycle on the Durability of Cement Concrete. Buildings.

[13] He B., Xie M., Jiang Z., Zhang C., Zhu X. (2020). Temperature Field Distribution and Microstructure of Cement-Based Materials under Cryogenic Freeze-Thaw Cycles. Constr. Build. Mater..

[14] Li Y., Liu Y., Guo H., Li Y. (2024). Investigation of the Freeze-Thaw Deterioration Behavior of Hydraulic Concrete under Various Curing Temperatures. J. Build. Eng..

[15] Sun G., Wang N., Yao Y. (2026). Analysis of Moisture Migration Patterns in Pavement Concrete Induced by the Pot Cover Effect. Transp. Geotech..

[16] Zhao N., Lian S. (2024). Study of Damage Mechanism and Evolution Model of Concrete under Freeze–Thaw Cycles. Appl. Sci..

[17] Shang H.-S., Yi T.-H., Song Y.-P. (2012). Behavior of Plain Concrete of a High Water-Cement Ratio after Freeze-Thaw Cycles. Materials.

[18] Sicat E., Gong F., Ueda T., Zhang D. (2014). Experimental Investigation of the Deformational Behavior of the Interfacial Transition Zone (ITZ) in Concrete during Freezing and Thawing Cycles. Constr. Build. Mater..

[19] Song H., Yao J., Xiang J. (2022). The Role of Aggregate and Cement Paste in the Deterioration of the Transitional Interface Zone of Pervious Concrete during Freeze-Thaw Cycles. Case Stud. Constr. Mater..

[20] Mohammed I., Zhengfeng Z., Changfa A. (2024). Evaluating the Impact of Temperature Variations and Subgrade Reactions under Traffic-Load on Airport Concrete Pavement Performance. Structures.

[21] Mohamed A.R., Hansen W. (1996). Prediction of Stresses in Concrete Pavements Subjected to Non-Linear Gradients. Cem. Concr. Compos..

[22] Yu S., Huang Y., Liu Z., Long Y. (2025). Compressive-Shear Behavior and Cracking Characteristics of Composite Pavement Asphalt Layers Under Thermo-Mechanical Coupling. Materials.

[23] Liu Q., Pei J., Wang Z., Hu D., Huang G., Meng Y., Lyu L., Zheng F. (2024). Analysis of Tire-Pavement Interaction Modeling and Rolling Energy Consumption Based on Finite Element Simulation. Constr. Build. Mater..

[24] Kane M., Lim M., Tan Do M., Edmondson V. (2022). A New Predictive Skid Resistance Model (PSRM) for Pavement Evolution Due to Texture Polishing by Traffic. Constr. Build. Mater..

[25] (2009). Standard for Test Methods of Long-Term Performance and Durability of Ordinary Concrete.

[26] Xu H., Zhong K., Zhou S., Qiu H., Sun M. (2026). Mechanical Response Characteristics of Concrete Runway Under Aircraft Impact Loadings Based on Tire-Pavement-Temperature Coupled Model. Int. J. Pavement Res. Technol..

[27] (2025). Specifications for Airport Cement Concrete Pavement Design.

[28] (2024). Specifications for Construction of Aerodrome Cement Concrete Pavement.

[29] (2010). Code for Design of Concrete Structures.

[30] Zhang H., Goltsberg R., Etsion I. (2022). Modeling Adhesive Wear in Asperity and Rough Surface Contacts: A Review. Materials.

[31] Liu X., Li J., Liu J., Huang C., Liu L. (2024). Prediction of Permanent Deformation of Subgrade Soils under F-T Cycles Using SABO-Optimized CNN-BiLSTM Network. Case Stud. Constr. Mater..

[32] Nadour M., Rabehi A., Hadroug N., Guermoui M., Tibermacine I.E., Alanazi A.K., Habib M., Rabehi A. (2026). Deep Hybrid CNN–biLSTM Model for Accurate Solar Photovoltaic Power Forecasting: A Comparative Study with Classical and Neural Models. Energy Rep..

[33] Nohara Y., Matsumoto K., Soejima H., Nakashima N. (2022). Explanation of Machine Learning Models Using Shapley Additive Explanation and Application for Real Data in Hospital. Comput. Methods Programs Biomed..

[34] Meng Q., Zhong K., Sun M. (2023). Dynamic Response Analysis of Airport Pavement under Impact Loading. Appl. Sci..

[35] Liu S., Ling J., Tian Y., Hou T. (2024). Evaluation of Aircraft Random Vibration under Roughness Excitation during Taxiing. Int. J. Transp. Sci. Technol..

[36] Liu J., Zhou D., Cheng L., An S., Guo L., Xue H., Wu R. (2024). Analysis of Damage and Fracture Characteristics for Concrete Subjected to Cryogenic Freeze-Thaw Cycles: An Acoustic Emission and Digital Image Correlation Study. J. Build. Eng..

